# Linear growth faltering in infants is associated with *Acidaminococcus* sp. and community-level changes in the gut microbiota

**DOI:** 10.1186/s40168-015-0089-2

**Published:** 2015-06-13

**Authors:** Ethan K. Gough, David A. Stephens, Erica E.M. Moodie, Andrew J. Prendergast, Rebecca J. Stoltzfus, Jean H. Humphrey, Amee R. Manges

**Affiliations:** Department of Epidemiology, Biostatistics and Occupational Health, McGill University, Montreal, H3A 1A2, QC Canada; Department of Mathematics and Statistics, McGill University, Montreal, H3A 2K6, QC Canada; Centre for Paediatrics, Blizard Institute, Queen Mary University of London, London, E1 2AT UK; Zvitambo Institute for Maternal Child Health Research, Harare, Zimbabwe; Program in International Nutrition, Division of Nutritional Sciences, Cornell University, Ithaca, NY 14853 USA; Department of International Health, Johns Hopkins Bloomberg School of Public Health, Baltimore, MD 21205 USA; Faculty of Medicine, School of Population and Public Health, University of British Columbia, 137-2206 East Mall, Vancouver, V6T 1Z3, BC Canada

**Keywords:** Microbiota, Microbiome, Intestinal, Stunting, Growth, Statistical learning, Networks

## Abstract

**Background:**

Chronic malnutrition, termed stunting, is defined as suboptimal linear growth, affects one third of children in developing countries, and leads to increased mortality and poor developmental outcomes. The causes of childhood stunting are unknown, and strategies to improve growth and related outcomes in children have only had modest impacts. Recent studies have shown that the ecosystem of microbes in the human gut, termed the microbiota, can induce changes in weight. However, the specific changes in the gut microbiota that contribute to growth remain unknown, and no studies have investigated the gut microbiota as a determinant of chronic malnutrition.

**Results:**

We performed secondary analyses of data from two well-characterized twin cohorts of children from Malawi and Bangladesh to identify bacterial genera associated with linear growth. In a case-control analysis, we used the graphical lasso to estimate covariance network models of gut microbial interactions from relative genus abundances and used network analysis methods to select genera associated with stunting severity. In longitudinal analyses, we determined associations between these selected microbes and linear growth using between-within twin regression models to adjust for confounding and introduce temporality. Reduced microbiota diversity and increased covariance network density were associated with stunting severity, while increased relative abundance of *Acidaminococcus* sp. was associated with future linear growth deficits.

**Conclusions:**

We show that length growth in children is associated with community-wide changes in the gut microbiota and with the abundance of the bacterial genus, *Acidaminococcus*. Larger cohorts are needed to confirm these findings and to clarify the mechanisms involved.

**Electronic supplementary material:**

The online version of this article (doi:10.1186/s40168-015-0089-2) contains supplementary material, which is available to authorized users.

## Background

Undernutrition in early childhood underlies 45 % of mortality in children aged under 5 years worldwide, resulting in 3.1 million deaths annually [1]. Ponderal and linear growth faltering in children are viewed as indicators of acute and chronic malnutrition, respectively, and are often measured in terms of *z*-scores (i.e., deviations in attained growth from a reference population median). Children whose length- or height-for-age *z*-scores (LAZ or HAZ) is more than 2 standard deviations below the reference population median are termed stunted. Stunting has short-term effects on morbidity and mortality [2], leads to poor motor development and cognition, and reduces educational and economic attainment over the life-course [1–3]. An estimated 165 million children under 5 years old were stunted in 2011 [1], representing almost one third of children in this age group in low- and middle-income countries (LMICs), hindering developmental potential and human capital of entire societies.

Most linear growth faltering occurs in the period from conception to 2 years of age, and restoration of deficits in linear growth beyond that period is limited. Interventions to prevent stunting are therefore required early in the life-course. Social, economic, and educational factors, as well as infectious diseases and poor diet in early childhood all contribute to linear growth faltering [1, 4–7]. Furthermore, a number of studies have shown that small intestinal inflammation and permeability are associated with poor linear growth [8–11]. This subclinical gut pathology has been termed environmental enteric dysfunction (EED) and is acquired early in life among children living in unsanitary conditions [5, 12–15]. Reduced intestinal barrier function caused by EED enables bacterial translocation to occur, leading to chronic systemic inflammation, which is associated with reduced insulin-like growth factor 1 (IGF-1) and linear growth faltering [16]. However, the pathophysiology of stunting is not well understood, and currently available interventions, which focus mostly on dietary supplementation and prevention of diarrhea, have only a modest impact [17]. Mechanisms underlying stunting therefore need to be better defined so that tractable pathways for intervention can be identified.

Recent studies suggest a role of the intestinal microbiota in child growth. The intestinal microbiota is an ecosystem of gut microbes that helps to modulate nutrient harvesting from the diet, mucosal inflammation, and the immune response in the gut [18–22]. Observational studies in humans [23–26] have demonstrated a relationship between the intestinal microbiota and severe acute malnutrition (SAM). A causal effect of the intestinal microbiota on weight has also been shown using experimental animal models [27, 28]. However, the specific changes in the microbiota that contribute to growth remain unclear, and no studies to date have investigated the intestinal microbiota as a determinant of linear growth.

We performed a secondary analysis of publicly available data from two twin cohorts of undernourished children from low-income settings (Malawi and Bangladesh) [25, 27], to identify bacterial genera whose relative abundances explain linear growth. Previous analyses from these cohorts showed that acute malnutrition was associated with differences in gut microbiota functional gene abundances [27] and maturation [25]. Our analyses aimed to determine changes in gut microbiota networks and relative abundance associated with stunting status, in order to identify potential microbiota members that contribute to linear growth faltering (i.e., chronic malnutrition). We hypothesized that differences in the relative abundance of identified genera are independently associated with prospective deficits in linear growth between siblings.

## Results and discussion

### Cohort description

Data were provided for 44 children in the Malawi cohort, who were median 10.2 months (interquartile range (IQR) 4.6, 14.5) old at baseline and followed for median 9.7 months (IQR 4.1, 14.5). Baseline HAZ and weight-for-height *z*-scores (WHZ) were −2.95 (IQR −3.70, −2.18) and −0.46 (IQR −0.87, −0.13), respectively. Anthropometric, epidemiological, and DNA whole genome shotgun sequencing data were provided for median 7 (IQR 4, 8) follow-up visits per child, for a total of 308 longitudinal observations (Additional file 1: Table S1). Data were available for 25 children in the Bangladesh birth cohort, who were 0.3 months (IQR 0.19, 0.63) old at baseline and followed for median 14.5 months (IQR 11.9, 20.7). Baseline HAZ and WHZ were −3.75 (IQR −4.54, −2.68) and −0.57 (IQR −1.51, 0.35), respectively. Anthropometric, epidemiological, and relative abundance data were provided for median 17 (IQR 13, 22) follow-up visits per child. Randomly excluding one child from the set of triplets for between-within regression analyses provided 429 longitudinal observations.

### Description of cases and controls

**Fig. 1:**
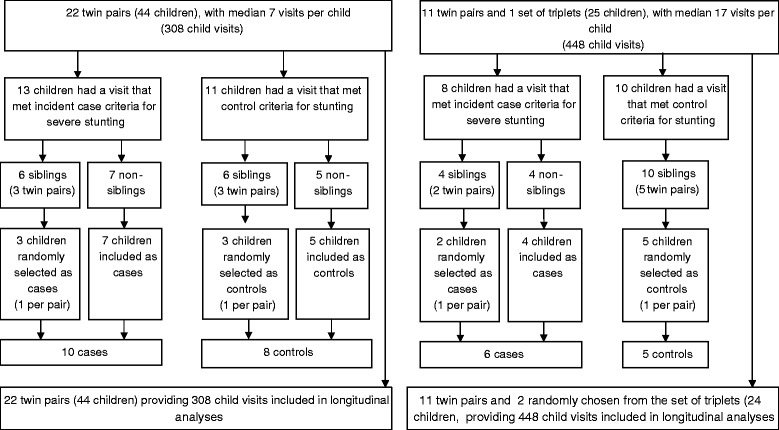
Flow chart of case and control selection from the Malawi twin cohort for network analysis (*left*) and flow chart of case and control selection from the Bangladesh twin cohort for network analysis (*right*)

In the Malawi cohort, 13 children had a follow-up visit that met incident case criteria for severe stunting, and 11 had a follow-up visit that met control criteria for stunting (see “Methods” for details on case and control definitions). Six eligible cases were co-twins, and six eligible controls were also co-twins. In the Bangladesh cohort, eight children had a follow-up visit that met incident case criteria, and ten had a follow-up visit that met control criteria. Four eligible cases were co-twins, and ten eligible controls were co-twins. For each pair of co-twins that both met case criteria, we randomly chose one sibling as a case to avoid within-group correlations [29]. The same was done for pairs of co-twins that both met control criteria. This provided ten cases and eight controls from Malawi, and six cases and five controls from Bangladesh (Fig. 1). Cases from the Malawi cohort had lower HAZ (−3.08 v −2.45, *p* < 0.01) and were younger compared to controls (10.8 v 19.6 months, *p* = 0.05). Similarly, in the Bangladesh cohort, case HAZ was −3.17 v −2.63 for controls, *p* < 0.01, and age was 2.9 v 11.0 months, *p* < 0.01. WHZ was also higher in Bangladesh cases compared to controls (0.53 v −0.64, *p* = 0.05) (Additional file 1: Table S1).

### Genus relative abundance and microbiota diversity

Roche 454 shotgun whole genome sequence data were provided for median 76,700 (IQR 55,200, 103,000) reads per sample in the entire Malawi cohort, while relative abundance data from the Bangladesh cohort were quantified from a median 20,192 (IQR 16, 155, 24,632) reads. In both cohorts, a similar number of reads were available for cases and controls (Additional file 1: Table S1).

In the Malawi cohort, *Bifidobacterium* (42.8 %) and *Prevotella* (22.7 %) were the most abundant genera identified, followed by *Bacteroides* (3.7 %), *Faecalibacterium* (3.14 %), *Collinsella* (1.0 %), *Lactobacillus* (0.6 %), and *Blautia* (0.6 %). In the Bangladesh cohort, *Bifidobacterium* (46.2 %), *Streptococcus* (4.8 %), *Lactobacillus* (2.6 %), and *Escherichia*/*Shigella* (1.8 %) were the most abundant genera, followed by *Collinsella* (0.5 %). These were also the most prevalent genera identified in fecal samples collected during follow-up (Additional file 2: Table S2) and are consistent with the literature on microbiota in infants and with different diets [30–35]. In the Malawi cohort, *Prevotella* (18.1 v 42.9, *p* = 0.06), *Bacteroides* (1.9 v 7.4, *p* = 0.01), *Eubacterium* (0.0 v 2.4, *p* < 0.01), and *Blautia* (0.6 v 2.4, *p* = 0.03) showed the largest decrease in relative abundance in cases v controls (Additional file 3: Table S3). In the Bangladesh cohort, *Lactobacillus* (0.1 v 8.7, *p* < 0.01), *Olsenella* (0.0 v 0.8, *p* < 0.01), *Dorea* (0.0 v 0.7, *p* = 0.05), *Blautia* (0.0 v 0.2, *p* < 0.01), and unclassified genera in the *Coriobacteriaceae* (0.0 v 0.3, *p* < 0.01) and *Enterococcaceae* (0.0 v 0.1, *p* = 0.08) families showed the largest decrease in relative abundance in cases v controls. Lesser, but statistically significant depletion of *Anaerococcus*, *Dialister*, *Faecalibacterium*, *Megamonas*, *Weissella*, *Megasphaera*, and unclassified genera in the *Lachnospiraceae*, *Lactobacillaceae*, and *Veillonellaceae* families were also observed in Bangladesh cases (Additional file 4: Table S4). Case microbiota were less diverse than controls in both cohorts (Malawi: 0.5 v 0.7, *p* = 0.02; Bangladesh: 0.5 v 0.7, *p* = 0.05) (Additional file 1: Table S1).

### Network indices

**Fig. 2:**
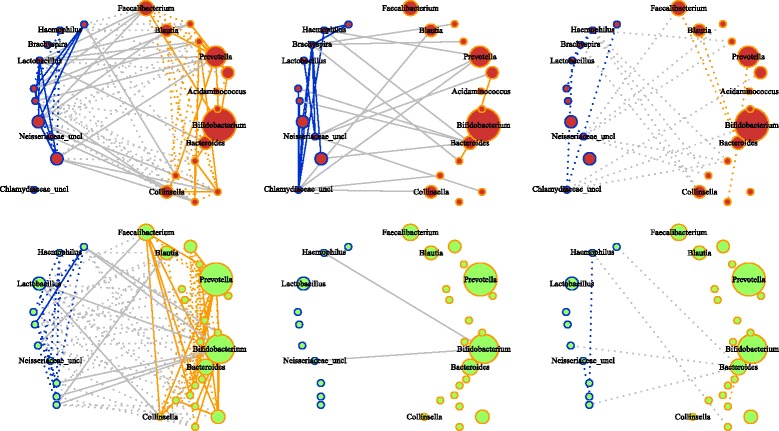
Graphical models of Malawi case and control microbiota networks constructed using glasso. (*Top*) Case networks. (*Bottom*) Control networks. (*Left to right*) Associations found in both groups, cases only and controls only. *Solid* and *dotted* edges indicate positive and negative associations. *Blue* indicates associations among aerobic and facultative anaerobic genera. *Orange* indicates associations among anaerobic genera. *Gray* indicates associations from aerobic/facultative anaerobic to anaerobic genera. Node size is proportional to median abundance

**Fig. 3:**
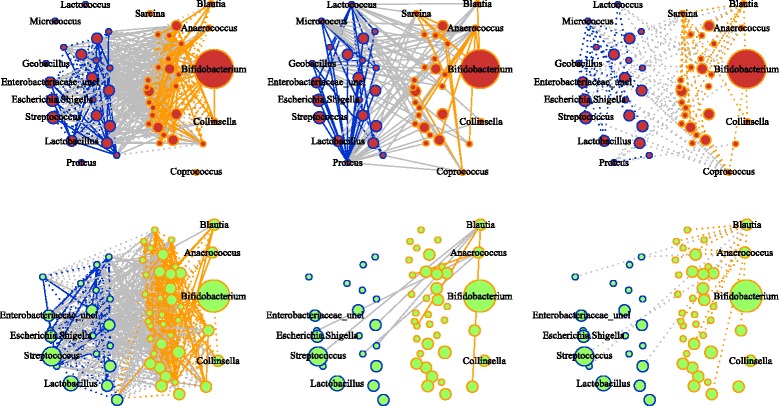
Graphical models of Bangladesh case and control microbiota networks constructed using glasso. (*Top*) Case networks. (*Bottom*) Control networks. (*Left to Right*) Associations found in both groups, cases only and controls only. *Solid* and *dotted* edges indicate positive and negative associations. *Blue* indicates associations among aerobic and facultative anaerobic genera. *Orange* indicates associations among anaerobic genera. *Gray* indicates associations from aerobic/facultative anaerobic to anaerobic genera. Node size is proportional to median abundance

Network density (i.e., the probability that two randomly selected microbes co-vary) was greater in case compared to control networks in both cohorts (Malawi: 0.56 v 0.25, *p* = 0.08; Bangladesh: 0.56 v 0.33, *p* = 0.42), indicating a greater potential for information flow in case microbiota. We also observed that the density of edges from aerobes to anaerobes was greater in the case network in both populations (Figs. 2 and 3).

In the Malawi cohort, differences in degree centrality were observed for *Acidaminococcus* (0.6 v 0.0, *p* = 0.06), *Bacteroides* (0.6 v 0.2, *p* = 0.03), *Brachyspira* (0.6 v 0.0, *p* = 0.09), *Haemophilus* (0.6 v 0.2, *p* = 0.07), and unclassified genera in the *Neisseriaceae* (0.6 v 0.2, *p* = 0.08) and *Chlamydiaceae* (0.6 v 0.0, *p* = 0.05) families in case v control networks (Additional file 3: Table S3). In the Bangladesh cohort, *Acinetobacter* (0.5 v 0.0, *p* = 0.03), *Anaerococcus* (0.7 v 0.2, *p* = 0.09), *Blautia* (0.7 v 0.2, *p* = 0.08), *Coprococcus* (0.5 v 0.0, *p* = 0.03), *Geobacillus* (0.6 v 0.0, *p* = 0.09), *Lactococcus* (0.6 v 0.0, *p* = 0.02), *Micrococcus* (0.5 v 0.0, *p* = 0.05), *Proteus* (0.6 v 0.0, *p* = 0.09), and *Sarcina* (0.6 v 0.0, *p* = 0.09) were more central in the case network (Additional file 4: Table S4).

### Between-within models

Thirty of 164 genera identified across both populations were selected, based on statistically significant differences in relative abundance or centrality, to estimate their association with future HAZ using multivariable between-within regression models. *Acidaminococcus*, of the phylum Firmicutes, was the only genus associated with HAZ in longitudinal analyses of both cohorts. In the Malawi cohort, a 0.1 % difference in the relative abundance of this genus between co-twins was associated with a 0.08 lower height-for-age *z*-score (90 % confidence interval (CI) −0.12, −0.04) at the subsequent study visit in the co-twin who had the greater *Acidaminococcus* abundance compared to their sibling. In the Bangladesh cohort, a 0.1 % difference in the relative abundance of this genus between co-twins was associated with a 0.19 lower HAZ (90 % CI −0.25, −0.13) at the subsequent visit in the co-twin with the greater *Acidaminococcus* abundance. These associations remained significant after controlling for multiple hypothesis testing (Table 1).

**Table 1:** Relative genus abundance associations with future HAZ estimated using multivariable between-within twin regression models for genera with a significant difference in degree centrality between cases and controls

	Malawi				Bangladesh			
Genus	Abundance difference^a^	Coefficient (90 % CI)	*p* value	Adjusted *p* value	Abundance difference^a^	Coefficient (90 % CI)	*p* value	Adjusted *p* value
*Acidaminococcus*	0.40	−0.080 (−0.124, −0.037)	<0.01	0.02	0.30	−0.191 (−0.253, −0.129)	<0.01	<0.01
*Acinetobacter* ^b^					0.00	−0.032 (−0.159, 0.094)	0.68	0.89
*Anaerococcus* ^b^					0.01	−0.182 (−0.915, 0.551)	0.68	0.89
*Bacteroides*	4.51	0.000 (−0.001, 0.001)	0.67	0.89	0.29	−0.001 (−0.002, 0.001)	0.63	0.89
*Blautia*	2.51	−0.001 (−0.003, 0.002)	0.64	0.89	5.00	0.001 (0.000, 0.001)	0.07	0.45
*Brachyspira*	1.03	0.003 (−0.002, 0.007)	0.32	0.89				
*Chlamydiaceae_uncl*	0.37	−0.012 (−0.054, 0.030)	0.65	0.89				
*Coprococcus*	0.35	−0.006 (−0.061, 0.049)	0.87	0.92	4.33	−0.003 (−0.010, 0.003)	0.38	0.89
*Geobacillus* ^b^					0.01	0.266 (−0.154, 0.685)	0.30	0.89
*Haemophilus*	0.76	0.001 (−0.009, 0.010)	0.92	0.92				
*Lactococcus* ^b^					0.04	−0.002 (−0.007, 0.004)	0.59	0.89
*Micrococcus* ^b^					0.46	−0.107 (−2.183, 0.169)	0.16	0.94
*Neisseriaceae_uncl*	0.22	−0.027 (−0.103,0.048)	0.56	0.89	0.01	0.001 (−0.001, 0.004)	0.46	0.64
*Proteus* ^b^					0.00	−0.002 (−0.037, 0.033)	0.94	0.94
*Sarcina* ^b^					5.00	0.000 (0.000, 0.001)	0.54	0.89

Coefficients are expressed as the average difference in future HAZ per 0.1 % difference in abundance between siblings

*90 % CI* 90 % confidence interval, *HAZ* height-for-age *z*-score

^a^Median difference in relative abundance between siblings in a twin pair

^b^Models could not be fit in the Malawi cohort because these genera were only identified in ≤2 samples

The literature on *Acidaminococcus* sp., with which we can infer its role in the human gut and its potential impact on linear growth in children, is sparse. Only two species in this genus have been described [36, 37]. One notable characteristic of these described species is their ability to consume glutamate as their sole source of carbon and energy. In porcine models, dietary glutamate is an essential oxidative fuel for the intestinal epithelium [38, 39], which undergoes a continuous process of regeneration and has high energy demands. Estimates for the amount of glutamate completely metabolized in the gut range from 64 [39] to 90 % [38]. As such, glutamate is important to gut epithelium restitution. The beneficial effect of glutamate on restoration of gut barrier function has been observed using *in vitro* cell lines [40–42], as well as in animal models of glutamate supplementation [43–46]. Glutamate is an important precursor and intermediate in the synthesis and metabolic recycling of other amino acids, and with the urea cycle, in the gut [38, 39, 47, 48]. Amino acids closely interlinked with glutamate metabolism include arginine, which also contributes to epithelium restitution, preserves barrier function, prevents accumulation of ammonia in the gut, and attenuates intestinal tissue damage [49–51], and glutathione, which protects the epithelium from damage by oxidative stress [52, 53]. Altogether, major functions of glutamate in the gut appear to be its role as a key intermediate in gut amino acid metabolism and nitrogen cycling, maintenance of epithelial integrity, and preservation of barrier function. Biomarkers of intestinal injury and repair have been associated with lower HAZ in LMICs [54]. Impaired gut barrier function is characteristic of EED, which is also associated with poor linear growth [8–11].

This evidence led us to pose the a posteriori hypothesis that glutamate fermentation by microbes is negatively associated with future HAZ. We tested this hypothesis using KEGG enzyme abundance data provided for the Malawi cohort. We fitted between-within regression models where the relative abundance of critical genes utilized in glutamate fermentation pathways by microbes [55] was the exposure of interest. We found that the abundance of genes encoding glutamate dehydrogenase and α-keto-glutarate reductase was negatively associated with future HAZ. For glutamate dehydrogenase and α-keto-glutarate reductase, respectively, a one unit greater gene abundance in one co-twin compared to their sibling was associated with a −0.17 (90 % CI −0.29, −0.04, *p* = 0.03) and −0.08 (90 % CI −0.16, −0.01, *p* = 0.07) smaller HAZ in that co-twin at the subsequent study visit. These are the first two enzymes involved in the hydroxyglutarate fermentation pathway used by *Acidaminococcus fermentans* for glutamate fermentation; some species in the *Peptoniphilus*, *Fusobacterium*, and Clostridia families can also utilize this pathway [55, 56].

In the Bangladesh cohort, we also observed a −0.003 (90 % CI −0.004, −0.002) lower HAZ and a 0.001 (90 % CI 0.000, 0.001) greater HAZ at the subsequent visit in co-twins who had a 0.1 % greater abundance of *Weissella* or *Blautia*, respectively, compared to their siblings (Table 1 and Additional file 5: Table S5). The association with *Blautia* was not statistically significant after controlling for multiple hypothesis testing.

### Discussion

In these analyses, we show that less diverse gut microbiota with greater covariance network density are associated with stunting severity, and an increase in the relative abundance of *Acidaminococcus* sp. is associated with lower future linear growth in two very different, well-characterized cohorts of children living in low-income settings. We applied a novel approach, utilizing a statistical learning method combined with network analysis and a permutation test to determine differences between microbiota communities of stunted and severely stunted children from these cohorts, and applied longitudinal epidemiological analysis methods to investigate whether changes in the genera identified were associated with future linear growth.

In our longitudinal models, greater abundance of *Acidaminococcus* was associated with a future deficit in HAZ between co-twins in both cohorts. *Acidaminococcus* sp. can utilize glutamate as their sole source of carbon and energy. Greater abundance of genes encoding the first two enzymes in the hydroxyglutarate pathway for glutamate fermentation was also associated with a future HAZ deficit. Overgrowth of bacteria that can ferment glutamate may have a deleterious effect on linear child growth, potentially as a result of glutamate’s importance in amino acid metabolism, nitrogen balance, and barrier function. This observation may also reflect the state of malnutrition in these cohorts of children, as the microbiota turns to host-associated proteins for energy. The weak negative association between *Weissella* and future HAZ observed in the Bangladesh cohort was not detected in the Malawi children and needs to be confirmed in other studies.

The impact of *Acidaminococcus* on growth may also involve its microbial relationships. Network analysis provides a useful framework for identifying important bacteria by their number of relationships [57–59]. One study used correlation network centrality measures to identify bacteria that successfully promote the growth conditions of a previously uncultivable microorganism [59]. In the Malawi cohort, *Acidaminococcus* showed a large increase in degree centrality in cases, indicating a potential increase in its influence on microbiota composition. The possibility that rare commensals can promote pathological states based on their relationships with other microbes, despite their low abundance, has been proposed [60] and is in line with the notion of keystone organisms [60–62]. Although an increase in *Acidaminococcus* centrality was not observed in the Bangladesh cases, random sampling error introduced by selecting cases and controls from such a small population (*n* = 25), lacking truly healthy control subjects of normal length, could bias how representative the case and control exposure histories were in that cohort. Larger epidemiological and experimental investigations are needed to confirm these findings and the mechanisms involved.

Finally, in both populations, we observed greater density in case networks that was only statistically significant in the Malawi cohort and a larger proportion of connections from aerobes to anaerobes in cases. An increase in the average number of connections with worsening nutritional status was also reported in children with SAM using correlation networks [23], and greater connectivity between aerobic and anaerobic bacteria was reported for the microbiota correlation network of children with moderate-to-severe diarrhea compared to non-diarrheal controls [63]. Simulation studies suggest that increased density may provide greater resource flow to nodes that are normally of low importance and may reduce the efficiency of resource flow out of the system [64, 65].

In construction of our graphical models, we adjusted for potential confounders that were reported (e.g., age and WHZ) but could not control for confounding when comparing case and control network indices. These differences may, therefore, still be confounded by age or by other unreported factors such as infant diet, maternal, or environmental variables, since controls were older than cases in both populations, and microbiota composition and structure may relate to the timing of complementary food introduction or environmental exposures. We cannot dismiss the possibility of spurious associations in our graphical models due to compositional effects [66], residual confounding by diet or other factors, and small sample size. The resulting “noise” limited our ability to detect differences between case and control networks, and we must exercise caution in interpreting pairwise associations as true ecological interactions.

The between-within multivariable regression models, however, control for unreported confounders that are shared between co-twins (e.g., fetal, maternal, and environmental), other factors that are identical between twins such as age at each visit and length of follow-up, as well as reported confounders that differ between siblings (e.g., diarrhea and infant sex). Data on any antibiotic use and diet at each visit were only provided for the Bangladesh cohort. Including antibiotic use and breastfeeding (without the use of formula or solid foods) in the between-within models did not change the results. The association between *Acidaminococcus* and linear growth was reproduced in *both* populations, suggesting that residual confounding due to other unreported factors that may differ between siblings, such as HIV status (these data were not available from either cohort), is unlikely. We also lagged these models so that changes in exposure preceded changes in growth. The temporality adds credibility to our main findings that an increase in *Acidaminococcus* and glutamate-fermenting microbes are associated with future growth deficits. Measurement error in quantification of relative abundance is unavoidable in microbiota studies. Since any such error is unlikely to be systematically related to *future* growth deficits *between* siblings, measurement error in these analyses would attenuate true associations with growth, further reducing our power in these small cohorts. Finally, the original cohort studies were not designed to investigate stunting. The average child in these populations already suffered from severe growth restriction at study entry, and these data may not elucidate the potential negative effect of microbiota dysbiosis or the protective effect of certain genera in children who are of normal length but still at risk of becoming stunted. This may apply particularly to the case-control analyses, for which there were no healthy, non-stunted controls.

## Conclusions

Our study applied a novel use of statistical learning and network methods to identify and interpret changes in graphical models of microbiota covariance patterns. They suggest that reduced microbiota diversity and changes in covariance network density are associated with stunting severity and that overgrowth of *Acidaminococcus*, and possibly other glutamate-fermenting microbes, may contribute to future growth deficits in already malnourished children. Our findings demonstrate the potential role that certain types of commensals in the gut may have on linear growth deficits. Larger primary studies in other settings, designed specifically to evaluate stunting in infants, are needed to confirm these findings, and experimental studies are needed to clarify the mechanisms involved.

## Methods

### Study sample

Demographic, clinical, and anthropometric data from a cohort of 22 twin pairs from Malawi, and a second cohort of 11 twin pairs plus one set of triplets from Bangladesh, were made available at http://gordonlab.wustl.edu/SuppData.html. Details are provided in Smith et al. [27] and Subramanian et al. [25]. In brief, 22 twin pairs ages birth to 3 years were selected from among 317 available pairs in five rural communities in Malawi for longitudinal analyses of their gut microbiota. Twin pairs were selected if at least four fecal samples were available from each co-sibling. The 12 sets of siblings from Bangladesh were selected from among mothers with multiple pregnancies at a child health and family planning clinic in Dhaka and were followed up for longitudinal gut microbiota evaluation. In both twin cohorts, at each follow-up visit, length/height and weight were measured, and fecal samples were collected along with data on age in months and diarrhea in the 7 days prior to or at the visit for Malawi and Bangladesh, respectively. Anthropometric measures were provided as height-for-age and weight-for-height *z*-scores. In the Malawi cohort, if at least one co-twin developed SAM, as defined using WHO criteria [67], both were treated with ready-to-use therapeutic food (RUTF).

### Whole genome sequencing and annotation

Whole genome sequence datasets from the Malawi cohort were made available through the European Bioinformatics Institute at http://www.ebi.ac.uk/ena/data/view/ERP001911&display=html [27] and MG-RAST (http://metagenomics.anl.gov/) [30]. Relative genus abundances (expressed as a percentage of the total amount of DNA assigned to a bacterial taxon in each stool sample) were estimated from shotgun reads using MetaPhlan [68]. Relative operational taxonomic unit (OTU) abundance data from the Bangladesh cohort were used as provided at http://gordonlab.wustl.edu/SuppData.html and were analyzed at the genus level. Extraction of genomic DNA from fecal samples, DNA sequencing, processing and filtering of reads, and, for Bangladesh data, OTU picking and taxon assignment have been described [25, 30]. The Simpson diversity index was calculated as a measure of alpha diversity in all samples using *vegan* [69]. Simpson diversity measures the probability that two randomly selected microbes in a sample will be from different taxa and provides a measure of the number of different types of bacteria present.

### Statistical analyses

Analyses were performed separately for the Malawi and Bangladesh cohorts using two approaches. We first conducted an analysis of unmatched cases and controls selected from each cohort in order to identify changes in microbiota networks and relative genus abundance associated with stunting status and to select genera for inclusion in longitudinal analyses. Next, in longitudinal analyses, we fitted multivariable regression models, using data available at all follow-up visits for the entire cohort of children, to control for confounding and to introduce temporality.

#### Case-control network analyses

Children in the Malawi and Bangladesh twin cohorts had median baseline HAZ of −2.96 (IQR −3.68, −2.18) and −3.75 (IQR −4.54, −2.68), respectively, indicating that the majority were severely stunted at study entry (Additional file 6: Figure S1). For the case-control analyses, linear growth status was therefore defined as severely stunted (HAZ ≤ −3, cases) or stunted (−3 < HAZ ≤ −2, controls). For cases, the visit where a child first reached HAZ ≤ −3 was selected, excluding children already severely stunted at study entry. The subset of children who were not siblings of cases and who had HAZ > −3 but ≤ −2 at the end of follow-up, regardless of their baseline *z*-score, was selected as controls. Spurious inferences can arise in the analysis of correlated data [70]. Since methods to address correlations are not available for the network analyses methods we implemented, if both siblings in a pair met case or control criteria, one was randomly chosen to avoid within-group correlations [29], and data from only one visit were used per child. Differences in anthropometric, demographic and epidemiological measures, alpha diversity, and relative abundance between cases and controls were evaluated using the chi-squared test or by permutation test on the median, as appropriate.

A supplemental approach to diversity indices for investigating the microbiota uses networks of pairwise correlations between taxa as a model of microbe-microbe interactions. In this representation, nodes are genera and a link between two nodes represents a non-zero association between two genera. This association is used as a proxy for bacterial interaction (see Additional file 7 for further information). An alternative to pairwise correlations is to estimate an inverse covariance matrix from genus abundances as a graphical model of important bacterial relationships. We generated these graphical models separately for cases and controls using the graphical lasso (glasso) [71]. The covariance associations estimated by the glasso (i.e., the links between genera in each network) are independent of all other taxa and covariates included in the model. For each case and control network, we calculated graph density and the normalized degree centrality of each genus [72] using *igraph* [73]. Differences in network indices were assessed for statistical significance by permutation. Specifically, children were randomly reallocated between the case and control groups 1000 times. For each permutation, one network was estimated per group and distributions of the difference in network indices between case and control networks were generated for statistical inference. Genera with significant differences in degree centrality or relative abundance between cases and controls were selected for longitudinal analyses.

#### Longitudinal analyses

After performing microbiota feature selection in the case-control analyses, we fitted between-within regression models [70, 74], using data for all follow-up visits from all twin pairs in each cohort (regardless of their selection as cases or controls), to investigate whether the relative abundance of selected genera was associated with linear growth. A between-within model allows estimation of the effect that differences in exposure level (e.g., differences in genus abundance) between siblings within a twin pair have on their outcomes, while adjusting for unmeasured confounders that siblings share, such as fetal, maternal, and environmental factors. This is done by including both (i) individual sibling exposure values and (ii) the mean exposure value of co-twins as covariates in a regression model. Adjustment for measured confounders not shared between co-twins (e.g., diarrhea) can be made by including sibling-specific covariates in the model [74].

We fitted a separate model for each genus selected, with relative abundance as the exposure and HAZ as the outcome. Each model was adjusted for reported diarrhea, WHZ, infant sex, and alpha diversity as reported confounders not shared by co-twins. Age in months and length of follow-up since baseline were also included as predictors of the outcome. All covariates were lagged by one visit in order to model their effect on future HAZ, with the exception of length of follow-up and age. All between-within models were fitted by generalized estimating equations (GEE) using *geepack* [75], and multiple hypothesis testing adjustments using the Benjamini-Hochberg method [76] were made. Statistical significance was determined at *α* < 0.1 due to the small sample size of both cohorts. All analyses were conducted in *R* version 3.0.1.

## Additional files

Additional file 1: Table S1. Study participant characteristics in each cohort at the baseline visit and in cases v controls. Summary statistics describing the baseline characteristics of participants enrolled in the Malawi and Bangladesh twin cohorts, and comparison of case versus control characteristics. Additional file 2: Table S2. Genus relative abundance and genus presence in 308 Malawi and 429 Bangladesh fecal samples collected during follow-up. Median, minimum and maximum relative abundance of each genus identified in 308 Malawi and 429 Bangladesh fecal samples collected during follow-up, and the number and proportion of samples in each cohort in which each genus was identified. Additional file 3: Table S3. Relative abundance and normalized degree centrality of genera identified in severely stunted cases and stunted controls selected from the Malawi cohort. Median, minimum and maximum relative abundance of each genus identified in 10 case or 8 control samples selected from the Malawi cohort. *P*-values were obtained by permutation test for a difference between case and control medians. Degree centralities of each genus identified in case or control samples were calculated from covariance network models of gut microbial interactions estimated using the graphical lasso, and were normalized for the number of genera in each network. *P*-values were obtained by permutation test for a difference between case and control degree centralities. Additional file 4: Table S4. Relative abundance and normalized degree centrality of genera identified in severely stunted cases and stunted controls selected from the Bangladesh cohort. Median, minimum and maximum relative abundance of each genus identified in 6 case or 5 control samples selected from the Bangladesh cohort. *P*-values were obtained by permutation test for a difference between case and control medians. Degree centralities of each genus identified in case or control samples were calculated from covariance network models of gut microbial interactions estimated using the graphical lasso and were normalized for the number of genera in each network. *P*-values were obtained by permutation test for a difference between case and control degree centralities. Additional file 5: Table S5. Relative genus abundance associations with future HAZ estimated using multivariable between-within twin regression models for genera with a significant difference in relative abundance between cases and controls. Associations between relative abundance and future HAZ for each genus with a statistically significant difference in median abundance between cases and controls selected from either the Malawi or Bangladesh cohorts. Coefficients measure the average difference in future HAZ between siblings within a pair of twins that is associated with each 0.1 % difference in relative abundance between siblings. Coefficients are also adjusted for infant sex, weight-for-height z-scores, diarrhea, and alpha diversity using multivariable between-within twin regression, since these factors may differ between co-twins. Additional file 6: Figure S1. Histograms of height-for-age z-score distributions in Malawi and Bangladesh children at study entry. Figure: (top) Height-for-age *z*-score distribution in the 44 Malawi children at study entry; (bottom) Height-for-age *z*-score distribution in the 25 Bangladesh children at study entry. Red vertical lines indicate the World Health Organization cut-off for stunting. Additional file 7.
**Extended Methods.** An extended description of the statistical analysis methods.
