# Erratum to: Linear growth faltering in infants is associated with *Acidaminococcus* sp. and community-level changes in the gut microbiota

**DOI:** 10.1186/s40168-016-0149-2

**Published:** 2016-01-22

**Authors:** Ethan K. Gough, David A. Stephens, Erica E.M. Moodie, Andrew J. Prendergast, Rebecca J. Stoltzfus, Jean H. Humphrey, Amee R. Manges

**Affiliations:** Department of Epidemiology, Biostatistics and Occupational Health, McGill University, Montreal, H3A 1A2 QC Canada; Department of Mathematics and Statistics, McGill University, Montreal, H3A 2K6 QC Canada; Centre for Paediatrics, Blizard Institute, Queen Mary University of London, London, E1 2AT UK; Zvitambo Institute for Maternal Child Health Research, Harare, Zimbabwe; Program in International Nutrition, Division of Nutritional Sciences, Cornell University, Ithaca, NY 14853 USA; Department of International Health, Johns Hopkins Bloomberg School of Public Health, Baltimore, MD 21205 USA; Faculty of Medicine, School of Population and Public Health, University of British Columbia, 137-2206 East Mall, Vancouver, V6T 1Z3 BC Canada

## Erratum to:

After publication of this article [1], the author noticed an error to Fig. 2 (Figure 1 here). The published Figure did not include the links involving the genus Acidaminococcus. The correct version of Fig. 2 (Figure 1 here) is included below.

**Fig. 1:**
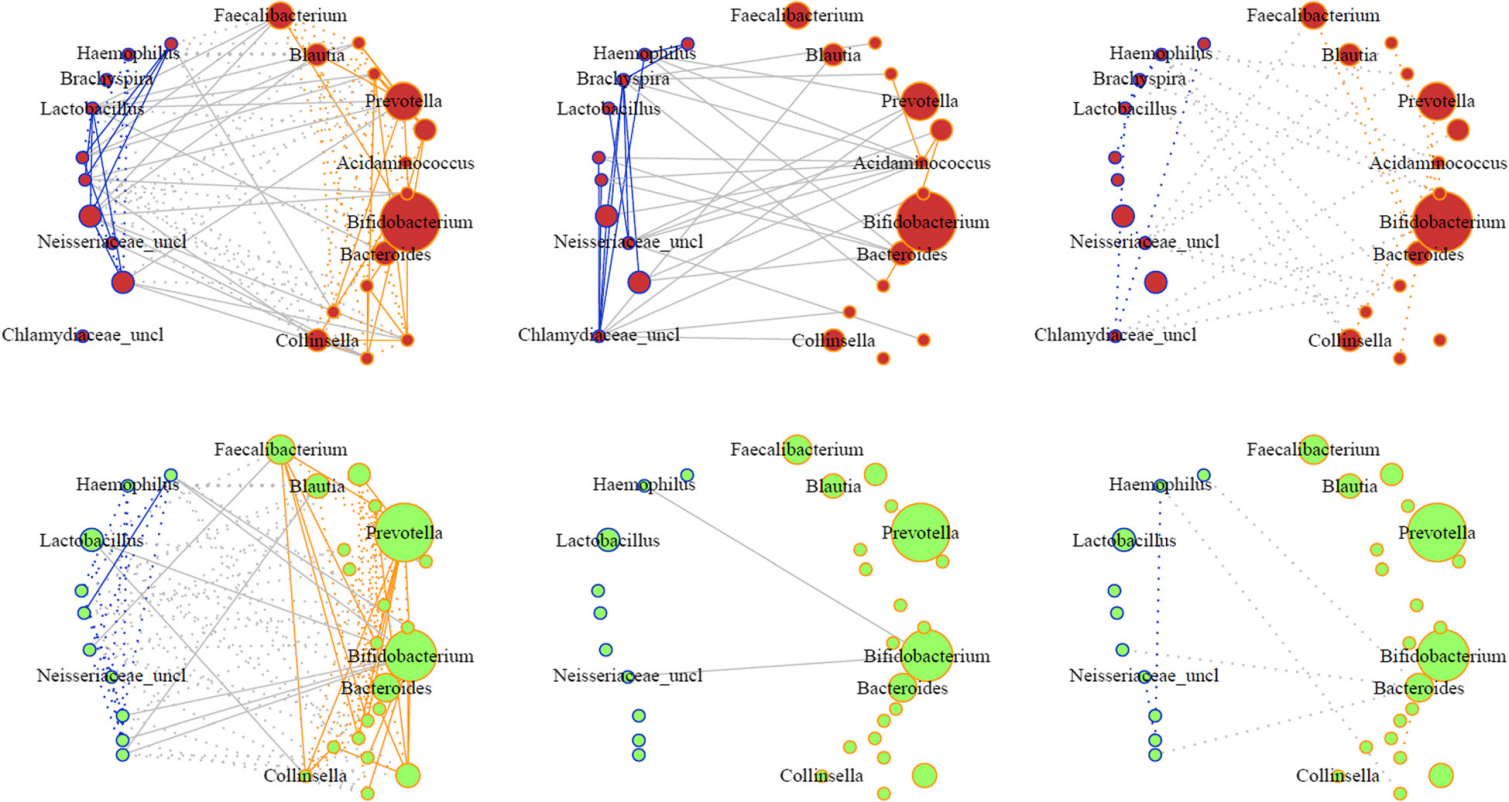
Graphical models of Malawi case and control microbiota networks constructed using glasso. (*Top*) Case networks. (*Bottom*) Control networks. (*Left to right*) Associations found in both groups, cases only and controls only. *Solid* and *dotted* edges indicate
