# Supplementary material for: Linear growth faltering in infants is associated with Acidaminococcus sp. and community-level changes in the gut microbiota
Source: Microbiome. 2015 Jun 13;3:24. doi: 10.1186/s40168-015-0089-2 (PMC4477476; doi:10.1186/s40168-015-0089-2)
Supplement: Additional file 5: Table S5. — Relative genus abundance associations with future HAZ estimated using multivariable between-within twin regression models for genera with a significant difference in relative abundance between cases and controls. Associations between relative abundance and future HAZ for each genus with a statistically significant difference in median abundance between cases and controls selected from either the Malawi or Bangladesh cohorts. Coefficients measure the average difference in future HAZ between siblings within a pair of twins that is associated with each 0.1 % difference in relative abundance between siblings. Coefficients are also adjusted for infant sex, weight-for-height z-scores, diarrhea, and alpha diversity using multivariable between-within twin regression, since these factors may differ between co-twins. [file 40168_2015_89_MOESM5_ESM.doc]

Additional file 5: Table S5. Relative Genus Abundance Associations with Future HAZ Estimated Using Multivariable Between-Within Twin Regression Models for Genera with a Significant Difference in Relative Abundance between Cases and Controls

|  | **Malawi** | | | | **Bangladesh** | | | |
| --- | --- | --- | --- | --- | --- | --- | --- | --- |
| **Genus** | **Abundance Difference^*^** | **Coefficient(90%CI)** | **p-value** | **Adjusted p-value** | **Abundance Difference^*^** | **Coefficient(90%CI)** | **p-value** | **Adjusted p-value** |
|
| Bifidobacteriaceae_uncl^†^ |  |  |  |  | 0.35 | 0.033(-0.078,0.143) | 0.63 | 0.92 |
| Coriobacteriaceae_uncl^†^ |  |  |  |  | 2.06 | -0.001(-0.005,0.004) | 0.82 | 0.92 |
| Dialister^†^ |  |  |  |  | 3.69 | -0.001(-0.004,0.003) | 0.73 | 0.92 |
| Dorea | 0.53 | -0.001(-0.010,0.008) | 0.91 | 0.91 | 3.54 | 0.002(0.000,0.004) | 0.10 | 0.58 |
| Enterococcaceae_uncl^†^ |  |  |  |  | 0.01 | 0.002(-0.011,0.014) | 0.81 | 0.92 |
| Eubacterium | 5.06 | 0.000(-0.001,0.000) | 0.48 | 0.78 | 4.10 | 0.002(-0.005,0.008) | 0.68 | 0.92 |
| Faecalibacterium | 5.47 | 0.000(-0.001,0.002) | 0.68 | 0.78 | 4.47 | 0.000(-0.002,0.002) | 0.97 | 1.00 |
| Lachnospiraceae_uncl^†^ |  |  |  |  | 4.33 | 0.000(-0.003,0.003) | 1.00 | 1.00 |
| Lactobacillaceae_uncl^†^ |  |  |  |  | 0.46 | -0.007(-0.043,0.030) | 0.77 | 0.92 |
| Lactobacillus | 7.25 | 0.000(-0.001,0.001) | 0.49 | 0.78 | 0.39 | 0.000(-0.001,0.001) | 0.71 | 0.92 |
| Megamonas | 1.96 | 0.002(-0.002,0.005) | 0.41 | 0.78 | 2.06 | -0.424(-9.736,8.887) | 0.94 | 0.92 |
| Olsenella^†^ |  |  |  |  | 0.50 | 0.000(0.000,0.001) | 0.37 | 0.92 |
| Prevotella | 12.48 | 0.000(-0.001,0.000) | 0.32 | 0.78 | 1.94 | 0.000(0.000,0.001) | 0.37 | 0.92 |
| Veillonellaceae_uncl^†^ |  |  |  |  | 3.21 | -0.001(-0.003,0.000) | 0.18 | 0.83 |
| Weissella^†^ |  |  |  |  | 0.46 | -0.003(-0.004,-0.002) | <0.01 | <0.01 |

Coefficients are expressed as the average difference in future HAZ per 0.1% difference in abundance between siblings. 90%CI: 90% confidence interval, HAZ: height-for-age z-score. ^*^Median difference in relative abundance between siblings in a twin pair. ^†^Models could not be fit in the Malawi cohort because these genera were only identified in ≤2 samples.
