# Supplementary figures and images for: Linear growth faltering in infants is associated with Acidaminococcus sp. and community-level changes in the gut microbiota
Source: Microbiome. 2015 Jun 13;3:24. doi: 10.1186/s40168-015-0089-2 (PMC4477476; doi:10.1186/s40168-015-0089-2)

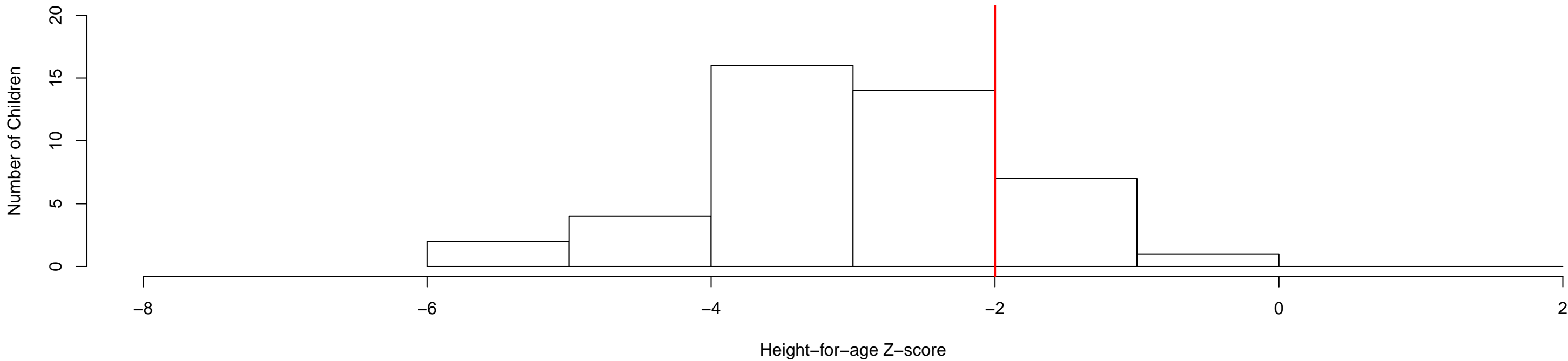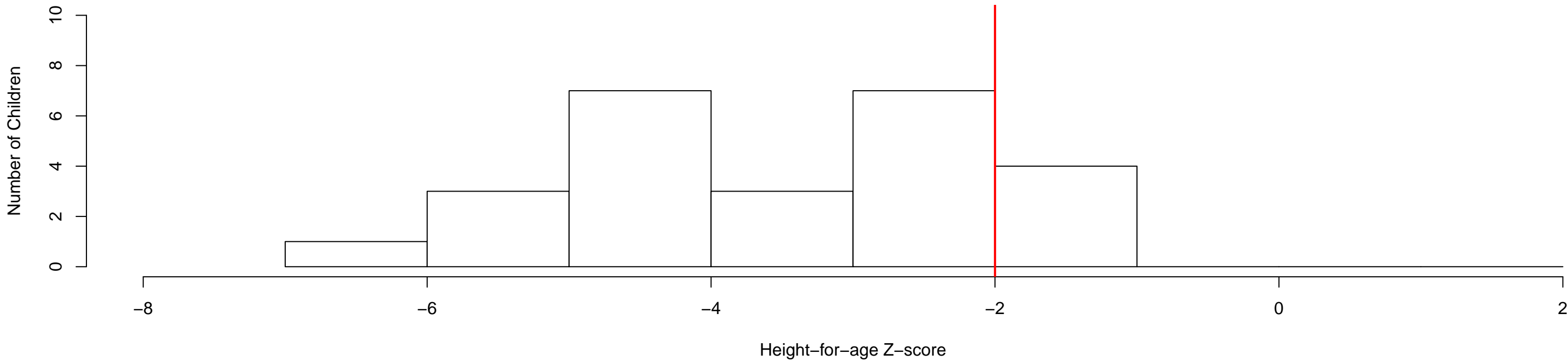

Supplement: Additional file 6: Figure S1. — Histograms of height-for-age z-score distributions in Malawi and Bangladesh children at study entry. Figure: (top) Height-for-age z-score distribution in the 44 Malawi children at study entry; (bottom) Height-for-age z-score distribution in the 25 Bangladesh children at study entry. Red vertical lines indicate the World Health Organization cut-off for stunting. [file 40168_2015_89_MOESM6_ESM.pdf]
