# Supplementary material for: Linear growth faltering in infants is associated with Acidaminococcus sp. and community-level changes in the gut microbiota
Source: Microbiome. 2015 Jun 13;3:24. doi: 10.1186/s40168-015-0089-2 (PMC4477476; doi:10.1186/s40168-015-0089-2)
Supplement: Additional file 7. — Extended Methods. An extended description of the statistical analysis methods. [file 40168_2015_89_MOESM7_ESM.doc]

**Additional data file 7: Extended Methods**

Case-Control Network Analyses

A supplemental approach to diversity indices for investigating the microbiota uses correlation networks as a model of microbe-microbe interactions. Microorganisms in the gut interact in a range of beneficial or antagonistic ecological relationships that arise, for example, through exchange of metabolic products or by-products, competition for nutrients, molecular signaling, or co-aggregation into consortia (1,2). Networks reflecting the pattern of co-occurrence between microbial taxa can be used as a model of bacterial ecological interactions (1,3). Use of such models is based on the premise that non-random patterns in taxon co-occurrence arise through such ecological relationships (4,5). These correlation network models of microbiota interactions have been used by some studies to date to investigate aspects of human microbiota community assembly (3) and its relationship to disease (6,7). We utilize an approach which estimates the covariance structure of abundance data as a graphical model of interactions (as opposed to statistical testing of pairwise correlations), based on the rationale that the covariance structure describes the microbial relationships that give rise to the observed distribution of abundances.

We estimated undirected graphical models from genus abundances, separately for cases and controls using the graphical lasso (glasso) (8). The glasso estimates an inverse covariance matrix from genus abundance data. Each pairwise value in the matrix is adjusted on the abundances of the remaining taxa in the microbiota, and any other covariates included. We included age and WHZ as additional covariates, as well as RUTF treatment in the Malawi data analyses. The algorithm also returns an estimated matrix where weak associations between taxa are shrunk to zero to ensure that insignificant dependences between taxa are excluded. We obtained one matrix for cases and one for controls. Each matrix was transformed into an unweighted, undirected network for subsequent network analyses. In this representation, nodes are genera, and a link between two nodes represents a non-zero association between two genera that is independent of all other taxa identified, age, WHZ, and RUTF treatment; this association is used as a proxy for bacterial interaction. To select the best tuning parameter for graphical model estimation, we used the Stability Approach for Regularisation Selection method (StARS) (9).

For each case and control network, we calculated graph density, and the normalized degree centrality of each taxon (10). Graph density is expressed as the probability (0 to 1) that two randomly selected nodes are connected, and provides a measure of the potential for information flow (e.g. nutrients, metabolic by-products, and molecular signals) over the network. Degree centrality provides a measure of node importance, based on assumptions regarding how information flows between nodes (11). Assuming that information can flow from a single node to multiple other microbes simultaneously, normalized degree (expressed as the proportion [0 to 1] of other genera that a specific genus is connected to) can be regarded as a measure of a microbe’s participation in information flow. A node with a larger number of connections can have greater influence in the network. It thus provides a measure of node importance that is useful for identifying members in a microbial community that can exert a disproportionate impact on its composition and function (10,12,13).

Differences in network indices were assessed for statistical significance by permutation test with 1000 randomizations. Specifically, children were randomly reallocated between the case and control groups 1000 times. For each permutation, one network was estimated per group, as described, and distributions of the difference in network indices between case and control networks were generated for statistical inference. Genera with significant differences in degree centrality or relative abundance between cases and controls were selected for longitudinal analyses.

Longitudinal Analyses to Test a posteriori Hypotheses

We tested our a posteriori hypothesis that glutamate fermentation by microbes is negatively associated with future HAZ using KEGG enzyme abundance data provided for the Malawi cohort at <http://gordonlab.wustl.edu/SuppData.html>. We fitted between-within regression models, using the relative abundance of genes encoding glutamate dehydrogenase (EC1.4.1.2), α-keto-glutarate reductase (EC1.1.99.2), and methylaspartate mutase (EC5.4.99.1) as exposures. These are critical enzymes in glutamate fermentation pathways used by microbes (14). We fitted a separate model for each gene, with relative abundance as the exposure and HAZ as the outcome. Each model was adjusted for reported diarrhea, WHZ, and alpha diversity as reported confounders not shared by co-twins. Age in months and length of follow-up since baseline were also included as predictors of the outcome. All covariates were lagged by one visit in order to model their effect on future HAZ, with the exception of length of follow-up and age. Functional gene abundance data were not available for the Bangladesh cohort because only the 16S gene was sequenced.

Reproducibility

Any bacterial genus that was found to have a significant difference in degree centrality or relative abundance between cases and controls in either cohort was investigated in both datasets as a determinant of future linear growth using multivariable between-within regression models. This allowed us to confirm any microbiota associations with future linear growth we identified.

**References**

1. Faust K, Raes J. Microbial interactions: from networks to models. *Nat Rev Microbiol* 2012; **10**:538–550.

2. Little AEF, Robinson CJ, Peterson SB, Raffa KF, Handelsman J Rules of Engagement: Interspecies Interactions that Regulate Microbial Communities. *Annu Rev Microbiol* 2008;**62**:375–401.

3. Faust K, Sathirapongsasuti JF, Izard J, Segata N, Gevers D, Raes J, *et al.* Microbial Co-occurrence Relationships in the Human Microbiome. *PLoS Comput Biol* 2012 **8**:e1002606.

4. Jeraldo P, Sipos M, Chia N, Brulc JM, Dhillon AS, Konkel ME, *et al.* Quantification of the relative roles of niche and neutral processes in structuring gastrointestinal microbiomes. *Proc Natl Acad Sci* 2012; **109**:9692–9698.

5. Wootton JT. Field parameterization and experimental test of the neutral theory of biodiversity. *Nature* 2005; **433**:309–312.

6. Endesfelder D, zu Castell W, Ardissone A, Davis-Richardson AG, Achenbach P, Hagen M. *et al.* Compromised gut microbiota networks in children with anti-islet cell autoimmunity. *Diabetes* 2014; **63**:2006–2014.

7. Ghosh TS, Gupta SS, Bhattacharya T, Yadav D, Barik A, Chowdhury A, *et al.* Gut Microbiomes of Indian Children of Varying Nutritional Status. *PLoS ONE* 2014; **9**:e95547.

8. Friedman J, Hastie T, Tibshirani R. Sparse inverse covariance estimation with the graphical lasso. *Biostatistics* 2008; **9**:432–441.

9. Liu H, Roeder K, Wasserman, L. Stability approach to regularization selection (stars) for high dimensional graphical models. *Advances in Neural Information Processing Systems* 2010; **23**:1432–1440.

1. Newman M. Measures and metrics. *Networks: an introduction*. Oxford: New York; 2010.

11. Borgatti SP. Centrality and network flow. *Soc Netw* 2005; **27**:55–71.

12. Hajishengallis G, Darveau RP, Curtis MA. The keystone-pathogen hypothesis. *Nat Rev Microbiol* 2012; **10:**717–725.

13. Ze X Le, Mougen F, Duncan SH, Louis P, Flint HJ. Some are more equal than others: The role of ‘keystone’ species in the degradation of recalcitrant substrates. *Gut Microbes* 2013; **4**:236–240.

14. Buckel W, Barker HA. Two pathways of glutamate fermentation by anaerobic bacteria. *J Bacteriol* 1974; **117**:1248–1260.
